# The Magee Equation: A Potential Economic Alternative in the Prognostication of Patients With Breast Cancer

**DOI:** 10.7759/cureus.97473

**Published:** 2025-11-21

**Authors:** Dhivya Yuvashree Subramanian, Vidhya Lakshmi

**Affiliations:** 1 Pathology, Rajalakshmi Medical College and Hospital, Chennai, IND; 2 Pathology and Laboratory Medicine, PSG (P.S. Govindasamy) Institute of Medical Sciences and Research, Coimbatore, IND

**Keywords:** clinical treatment score at 5 years (cts5), estrogen receptor (er), human epidermal growth factor receptor 2 (her2), oncotype dx recurrence score (odx rs), progesterone receptor (pr)

## Abstract

Introduction: Breast cancer is a heterogeneous disease classified by histopathological and molecular features. While the Oncotype DX assay is widely used to predict prognosis in estrogen receptor (ER)-positive, human epidermal growth factor receptor 2 (HER2)-negative cases, its high cost limits accessibility. The Magee equations, derived from immunohistochemical and clinicopathological variables, offer a cost-effective alternative for recurrence risk estimation. The Clinical Treatment Score at 5 years (CTS5) model further aids in predicting late distant recurrence using routine clinical parameters.

Aim and objectives: To derive the Magee Equation 3 (ME3) and CTS5 scores in each patient and categorize them into prognostic groups. To compare the ME3 score with the CTS5 score and observe the treatment response/recurrence rate in the study population. To hypothesize the validity and utility of the ME3 score as a cheaper alternative to more expensive genetic tests.

Materials and methods: A cross-sectional study of 50 consecutive cases of primary breast cancer, which are ER/progesterone receptor (PR) positive, has been included in the study. The CTS5 and ME3 recurrence score (RS) were calculated using the online websites https://cts5-calculator.com and https://path.upmc.edu/onlineTools/mageeequations.html, respectively. The results were tabulated. The follow-up records of patients were reviewed.

Results: A total of 50 cases of breast carcinoma were included. The number of cases in low-, intermediate-, and high-risk categories of the CTS5 score was 26 cases (52%), 19 cases (36%), and five cases (10%), respectively. The number of cases in low-, intermediate-, and high-risk categories of ME3 RS was two cases (4%), 31 cases (62%), and 17 cases (34%), respectively. A high level of concordance between ME3 RS and CTS5 scores was observed in the intermediate category (20%; n = 10).

Conclusion: The Magee Equation 3 (ME3) is a reliable, simple, and cost-effective tool that correlates well with the CTS5 model, making it a practical alternative for predicting recurrence risk and guiding treatment decisions in breast cancer.

## Introduction

Breast cancer exhibits significant morphological and genetic diversity, and its classification based on histologic type and grade has been refined through molecular assays that evaluate genomic profiles to aid in risk assessment and prognosis prediction [1]. According to the American Society of Clinical Oncology guidelines, the 21-gene recurrence score Oncotype DX (ODX; Exact Sciences, Madison, USA) can be used to inform adjuvant chemotherapy decisions in patients with estrogen receptor (ER)-positive, human epidermal growth factor receptor 2 (HER2)-negative breast cancer [2]. Oncotype Dx is time-consuming, and it causes a huge economic impact on our patients, so the quest for a cheaper yet reliable and validated alternative becomes essential [2].

The three new Magee equations were developed based on different hypotheses and available data. The first regression model (ME1) includes all parameters (including the Ki-67 index) for predicting Oncotype DX recurrence score (RS). The second regression model (ME2) was similar to ME1 but did not include Ki-67. The third regression model (Magee Equation 3 (ME3)) included semi-quantitative immunohistochemical expression levels for ER, progesterone receptor (PR), HER2, and Ki-67 [3]. Among all Magee equations, the Magee Equation 3 (ME3) was chosen for this study, as previous research has demonstrated that ME3 provides predictive accuracy comparable to Magee Equations 1 and 2 in estimating Oncotype DX recurrence scores. Moreover, ME3 offers superior reproducibility and practical applicability, making it particularly suitable for use in low-resource settings [3].

The Clinical Treatment Score at 5 years (CTS5) is a prognostic model developed from the combined Assay for Transposase-Accessible Chromatin (ATAC) and Breast International Group (BIG) 1-98 trial data to estimate the risk of distant recurrence in postmenopausal women with ER-positive breast cancer after completing five years of endocrine therapy [4]. It incorporates tumor size, nodal involvement, tumor grade, and age at diagnosis to calculate the five- to 10-year recurrence risk, classifying patients into low-, intermediate-, or high-risk groups accordingly [4]. The study compares the Magee Equation score with the CTS and observes the treatment response/recurrence rate in the study population. And to hypothesize the validity and utility of the Magee equation score as a cheaper alternative to more expensive genetic tests (specifically the Oncotype DX assay).

## Materials and methods

A cross-sectional (retrospective) study for which study material was derived from 50 consecutive cases of primary breast cancer, which are ER/PR positive, HER2 neu negative (luminal A and B subtypes), diagnosed during five years, was selected from the histopathology archives. Ethics approval was obtained from the Institutional Human Ethics Committee, PSG Institute of Medical Sciences & Research (IMSR), Coimbatore, India (Project no. 19/349, dated: June 14, 2021). The clinical details regarding age, menopausal status, and clinical stage were obtained from hospital case records.

The histopathological diagnosis; size; tumor, node, and metastasis (TNM) stage; and status of lymph node metastasis of the tumor were recorded from the histopathological reports. Representative slides were reviewed to verify the type, grade, lympho-vascular invasion, and stage of the tumor. All immunohistochemical (IHC) assessments were performed by a single senior pathologist blinded to clinical outcomes to minimize interobserver variability. The IHC slides were critically reviewed to calculate the H-score for ER/PR and Ki-67. The Clinical Treatment Score (CTS) was calculated based on age, tumor size, grade, and number of lymph nodes with metastatic deposits using the online website https://cts5-calculator.com. The Magee Equation 3 recurrence score was calculated using the formula ME3 score = 24.30812 +ER H-Score*(-0.02177) + PR H-score*(-0.02884) + (0 for HER2 negative, 1.46495 for HER2 equivocal, 12.75525 for HER2 positive) + Ki-67*0.18649 at https://path.upmc.edu/onlineTools/mageeequations.html published by the Department of Pathology of University of Pittsburgh Medical Centre. The results were tabulated. The follow-up records of each patient were reviewed, and details regarding adjuvant chemotherapy, chemotherapy-related complications, recurrence, metastasis, and death were recorded.

Sample size

A total of 50 consecutive cases of primary breast cancer fulfilling the inclusion criteria were included in this cross-sectional retrospective study. The sample size was determined based on the availability of cases within the study period from July 2016 to June 2021 at the Department of Medical Oncology, PSG hospitals.

Inclusion criteria

Women with breast cancer received treatment at the Department of Medical Oncology, PSG hospitals, for a period of five years from July 2016 to June 2021. Patients' histopathological reports and case records could be accessed through the hospital information system and medical records. Cases having immunohistochemical marker reports of ER, PR, HER2 neu, and Ki67 were available.

Exclusion criteria

Patients who defaulted treatment or for whom IHC marker status was not available were excluded.

Statistical analysis

Statistical Package for Social Sciences (SPSS) version 20 (IBM SPSS Statistics (IBM Corp., Armonk, NY, USA, released 2011)) was used for statistical analysis. Data was entered into the Excel (Microsoft Corp., Redmond, USA) spreadsheet. Descriptive statistics of the explanatory and outcome variables were calculated by mean, standard deviation for quantitative variables, frequency, and proportions for qualitative variables. Pearson’s correlation was calculated, and scatter plots were drawn to calculate the correlation between the CTS5 and the Magee score. The level of significance was set at 5%.

## Results

Clinicopathological profile

A total of 50 histologically confirmed cases of breast carcinoma were analyzed based on the inclusion and exclusion criteria. Among these, 48 (96%) were females and two (4%) were males, with patients’ ages ranging from 39 to 77 years. The highest number of cases occurred in the 51- to 60-year age group (n = 15; 30%). All tumors were classified according to the WHO 2020 classification of breast tumors as invasive ductal carcinoma, which included histologic variants such as invasive ductal carcinoma of no special type (IDC-NST), mucinous carcinoma, mixed invasive ductal carcinoma, mucinous carcinoma with neuroendocrine differentiation, intraductal papillary carcinoma with focal invasion, and invasive micropapillary carcinoma. The mean tumor size was 3.5 cm, with a predominance of right-sided lesions and one bilateral case.

Using the modified Scarff-Bloom-Richardson (SBR) grading system, five cases (10%) were grade 1, 34 cases (68%) were grade 2, and 11 cases (22%) were grade 3, with grade 2 being the most frequent. Based on *AJCC* 8th edition TNM staging, 14 (28%) were stage I, 24 (48%) stage II, nine (18%) stage III, and three (6%) stage IV, with stage II being the most common. Six patients (12%) received neoadjuvant chemotherapy, primarily in stages II and III. Axillary lymph node metastasis was present in 29 cases (58%), with involvement ranging from 0 to 16 nodes.

Clinical Treatment Score at 5 years (CTS5) distribution

The Clinical Treatment Score at 5 years (CTS5) was computed based on tumor size, grade, nodal status, and patient age using the validated online calculator. The majority of patients (n = 26; 52%) were in the high-risk category, while 19 (38%) and five (10%) were in the intermediate- and low-risk groups, respectively (Table 1).

**Table 1:** Distribution of cases according to CTS5 risk category (n = 50) This table illustrates the proportion of patients stratified by CTS5 risk categories. The CTS5 score was calculated using tumor size, histologic grade, number of positive nodes, and patient age. The data demonstrate that the majority of patients belonged to the high‑risk category, implying a greater probability of late recurrence. CTS5: Clinical Treatment Score at 5 years.

CTS5 Risk Category	Frequency (n)	Percentage (%)
High	26	52.0
Intermediate	19	38.0
Low	5	10.0
Total	50	100.0

Relationship between CTS5 and age

The mean age of patients decreased slightly with increasing risk: low-risk (61.2 ± 12.9 years), intermediate-risk (58.7 ± 10.3 years), and high-risk (57.5 ± 10.2 years). The difference was not statistically significant (p = 0.76) (Table 2).

**Table 2:** Comparison between CTS5 risk score and age at diagnosis This table compares the mean age and standard deviation across CTS5 risk categories. An ANOVA test revealed no statistically significant variation (p > 0.05), indicating that age alone did not influence the CTS5 risk classification. CTS5: Clinical Treatment Score at 5 years. *Significance at p<0.05.

CTS5 Risk Category	N	Age		F Value	p-Value*
		Mean	Std. Dev		
Low	5	61.20	12.911	0.274	0.76
Intermediate	19	58.74	10.311		
High	26	57.54	10.222		
Total	50	58.36	10.354		

Magee Equation 3 (ME3) distribution

The Magee Equation 3 (ME3) was calculated using ER, PR, HER2, and Ki-67 expression data as per the University of Pittsburgh’s online tool. Cases with higher recurrence scores showed markedly lower ER and PR H-scores, indicating an inverse relationship between hormone receptor expression and recurrence potential (Table 3).

**Table 3:** Comparison between ER H-score and Magee Equation category This table demonstrates the relationship between estrogen receptor H‑score and Magee Equation risk groups. An inverse correlation is observed: tumors with higher ME3 scores tended to show lower ER H-scores. Statistical analysis was performed using ANOVA (p = 0.151). ER: estrogen receptor, ME3: Magee Equation 3. *Significance at p<0.05.

Magee Equation Category	N	ER H-score		F Value	p-Value*
		Mean	Std. Dev		
Low	17	260.00	35.000	1.972	0.151
Intermediate	31	236.61	60.130		
High	2	195.00	21.213		
Total	50	242.90	53.319		

Comparison between CTS5 and ME3 risk categories

Distribution of cases according to both scoring systems revealed that most patients were classified as high risk by CTS5 (52%) and as intermediate risk by ME3 (62%). Only a small proportion (4%) were categorized as high risk by ME3 (Table 4).

**Table 4:** Comparison of the distribution of cases according to CTS5 risk category and ME3 RS (n = 50) This table depicts the degree of overlap between CTS5 and ME3 categories. Although partial concordance exists in intermediate‑risk cases, statistical analysis by McNemar’s test confirms significant discordance (p = 0.001), highlighting that the two systems may provide complementary, rather than identical, prognostic insights. CTS5: Clinical Treatment Score at 5 years, ME3: Magee Equation 3, RS: recurrence score.

Category	CTS5 Risk Category		Magee Equation	
	Frequency (n)	Percentage (%)	Frequency (n)	Percentage (%)
High	26	52.0	2	4.0
Intermediate	19	38.0	31	62.0
Low	5	10.0	17	34.0
Total	50	100.0	50	100.0

Correlation and concordance between CTS5 and ME3

Cross-tabulation of CTS5 and ME3 risk categories demonstrated the highest concordance in the intermediate group (20%; n = 10), while discordance was more common in both the low- and high-risk categories. McNemar’s test indicated a statistically significant difference (p = 0.001) (Table 5).

**Table 5:** Correlation and concordance between ME3 Recurrence Score category and CTS5 risk category This table depicts the degree of overlap between CTS5 and ME3 categories. McNemar's test - 0.001. Although partial concordance exists in intermediate‑risk cases, statistical analysis by McNemar’s test confirms significant discordance (p = 0.001), highlighting that the two systems may provide complementary, rather than identical, prognostic insights. ME3: Magee Equation 3, CTS5: Clinical Treatment Score at 5 years.

CTS5 Risk Category	Magee Equation Category			Total
	Low	Intermediate	High	
Low	1	4	0	5
	2.0%	8.0%	0.0%	10.0%
Intermediate	8	10	1	19
	16.0%	20.0%	2.0%	38.0%
High	8	17	1	26
	16.0%	34.0%	2.0%	52.0%
Total	17	31	2	50
	34.0%	62.0%	4.0%	100.0%

Microscopic and immunohistochemical findings

**Figure 1:**
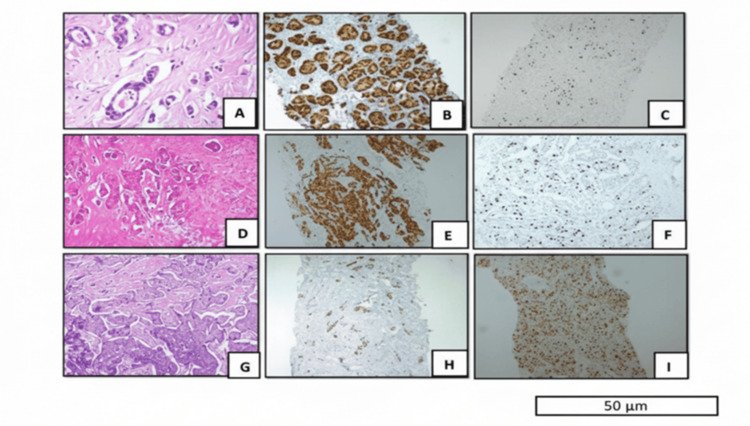
Representative histopathological and immunohistochemical (IHC) features across Magee Equation 3 risk categories Panels A-C: Low Magee score: Invasive carcinoma of no special type (NST), histologic grade 1 (hematoxylin and eosin (H&E) ×40); 90% of tumor cells expressing ER (IHC ×10) and 10%-20% of tumor cells expressing Ki‑67 (IHC ×10). Panels D-F: Intermediate Magee score: Invasive carcinoma NST, grade 2 (H&E ×10); 60%-70% of tumor cells expressing ER (IHC ×10) and 20%-30% of tumor cells expressing Ki‑67 (IHC ×10). Panels G-I: High Magee score: Invasive carcinoma NST, grade 3 (H&E ×10); 20%-30% of tumor cells expressing  ER (IHC ×10) and 70%-80% of tumor cells expressing Ki‑67 (IHC ×10). Scale bar = 50 µm. ER: estrogen receptor.

Microscopic and immunohistochemical illustrations demonstrated characteristic findings corresponding to each Magee Equation category: low-risk cases exhibited well-differentiated histology with strong ER positivity and low Ki-67 expression, whereas high-risk tumors showed poor differentiation, reduced ER expression, and high Ki-67 proliferation index (Figure 1).

Follow‑up outcomes

Among 50 cases, 39 cases (78%) had regular follow-up with a minimum period of 36 months. Seven cases (14%) had lost follow-up. Four cases (8%) had been declared dead, of which one case (2%) had recurrence.

## Discussion

Breast cancer is emerging as one of the curable malignancies, and there has been a tremendous explosion of knowledge about the pathogenesis and treatment strategies of breast cancer over the last two decades. Hormonal therapy with systemic chemotherapy and targeted therapy with monoclonal antibodies (trastuzumab or pertuzumab) have been the established adjuvant therapy for breast cancer [1]. The prognostic and predictive markers of breast cancers can be categorized as clinical (e.g., tumor size, lymph node status, stage), morphologic (size, grade, etc.), immunohistochemical (e.g., hormone receptors, Ki-67 labeling index), a single gene (HER2 neu), and multi-gene assays (Oncotype DX (Exact Sciences, Madison, USA); Mammaprint (Agendia, Amsterdam, Netherlands); EndoPredict (Myriad Genetics, Salt Lake City, USA); Prosigna (NanoString Technologies, Seattle, USA)). Oncotype DX is the most widely used and accepted molecular assay [2-4].

CTS5 score stratifies women into three distinctive risk groups: low, intermediate, and high categories. The CTS5 score includes the patient’s age, tumor size, tumor grade, and number of involved axillary nodes. In this study, 50 women with ER-positive breast cancer were considered. Of these, 13 patients were premenopausal and 37 patients were postmenopausal. The age of the patients varied between 39 and 77 years. The mean age of our patients was 55 years. The utility valve and validity of CTS5 score in breast cancer have been studied extensively by Wang et al., Tajiri et al., Lee et al., and Richman et al. [4-7]. CTS5 had a significant positive correlation with poor prognosis beyond five years in both postmenopausal and premenopausal subgroups [4]. In our study, the CTS5 score was calculated in all patients. Five patients had a low CTS5 score, 19 patients had an intermediate score, and 26 patients had a high score.

The advent of molecular assays has revolutionized precision medicine in breast cancer, with the TAILORx trial (2018) establishing the clinical utility of Oncotype DX, a 21‑gene recurrence score assay that aids in selecting patients for endocrine therapy alone or combined with chemotherapy, although its routine use remains limited by high cost and potential technical issues such as contamination from benign breast components and variability between tumor samples, while alternative algorithms like the Rochester Modified Magee Algorithm (RoMMa) have shown comparable predictive accuracy and offer a cost‑effective approach to molecular risk stratification [8].

In a large study, Tang et al. integrated the recurrence score with clinical and pathological factors and reported a better assessment of distant recurrence [9]. A study by Tang et al. provides evidence that standard clinical and pathological parameters can be even better than the Oncotype DX assay for prognostication [9]. Turner et al. (2015) published the original Magee Equation as a model to predict ODX recurrence score [10].

Klein et al. published an additional three Magee equations using a different combination of standard histopathological and IHC variables; the most recent Magee Equation 3 (ME3) is calculated from the H-score of ER/PR and Ki-67 proliferative index [3]. The H-score estimates the percentage of cells that are positive for each particular level of intensity (0-300) [10]. Magee Equations are user-friendly and available free of cost to the users. If an estimated Magee Equation recurrence score falls clearly in the high-risk or low-risk category, the oncologist should not expect a dramatically different ODX recurrence score from the estimated recurrence score [3].

Turner et al. indicated that the extreme Magee Equation scores (<9) or (>30) could accurately predict ODX recurrence scores and therefore save the cost of ODX by around 3 lakh rupees per case [10]. Turner et al (2015) undertook a multi-institutional validation of the algorithmic model and their results were that the Rochester Modified Magee algorithm correctly predicted 85% and 100% of cases to have a low and high ODX recurrence score [10].

In our study, we calculated the Magee Equation 3 score using the H-score for ER/PR and Ki-67 percentage. All the cases were categorized into three groups based on the scores. Seventeen patients belonged to a low category, 31 patients belonged to the intermediate category, and two patients belonged to the high category. The results of the Magee Equation were tabulated against the CTS5 score. Out of the 17 cases in the low category of the Magee Equation 3 score, only five cases belong to the low category of the CTS5 score. Out of 31 cases in the intermediate category of the Magee Equation 3 score, 19 cases belong to the intermediate category of the CTS5 score. Out of 26 cases in the high category of CTS5 score, only two cases belong to the high category of the Magee Equation 3 score. Our study is one of the first few studies to evaluate Magee Equation recurrence scores across various CTS5 categories. Bhargava et al. and a few other studies compared CTS5 risk model and 21-gene recurrence scores, yet an exhaustive literature search did not reveal similar studies involving comparison/correlation between the Magee Equation and CTS5 scores [11].

Pathologic complete response (pCR) rate after standard neoadjuvant chemotherapy in ER-positive/HER2-neu-negative cohort was limited to tumors with pretherapy Magee Equation scores >25 [12]. Farrugia et al. published a large single institutional study which showed a strong predictive value of Magee Equation 3 in the neoadjuvant setting as a standalone test. Saigosoom et al. evaluated the efficacy of the Magee Equation in predicting response and outcome in hormone-receptor-positive/HER2-neu-negative patients receiving neoadjuvant chemotherapy [13]. They found an association between high Magee Equation scores and pCR after neoadjuvant chemotherapy [12]. Only six out of 50 patients in our study received neoadjuvant chemotherapy. Hence, the pathological response to chemotherapy in various classes could not be studied.

Saigosoom et al. demonstrated a strong concordance between the Oncotype DX recurrence score and the modified Magee Equation in low-risk breast cancer, suggesting that the Magee Equation may serve as a reliable and cost-effective alternative for recurrence risk assessment [13]. Farrugia et al. reported that survival rates for low, intermediate, and high Magee Equation scores were 98%, 97%, and 79%, respectively [12]. Patients with high scores had a 10-fold increased risk of death [12]. The National Comprehensive Cancer Network guidelines recommend that Oncotype DX testing should be done in all low-grade invasive breast carcinomas which are hormonal receptors positive and HER2 neu negative; hence, Magee Equation may be an alternative method to stratify these low-grade invasive breast carcinomas [14]. In our study out of 50 patients, four (8%) patients died during treatment with recurrence in one patient. The Magee Equation score was high (>30%) among these patients. The rest of the patients were on regular follow-up. Bhargava et al. devised an algorithmic approach to withhold Oncotype DX testing in routine practice [15].

The finding of our study recommends the use of a modified Magee Equation in combination with clinical treatment score. In our study, there was no statistically significant correlation between CTS5 scores and Magee Equation scores. The p-value for concordance between the Magee Equation 3 recurrence score and CTS5 risk score was 0.418. This emphasizes the hypothesis that CTS5 and Magee Equation 3 scores are complementary and should always be used in combination as a predictive tool for prognosis/planning of treatment strategies. Magee Equations were developed as prognostic assays, but now they are frequently used as predictive assays, especially in the management of hormonal receptor-positive/HER2-negative breast cancers.

Limitations of the study

Preanalytical factors that can interfere with accurate immunohistochemical scoring were not considered in our study. The performance and reporting of the immunohistochemical assays can show some degree of interobserver and/or interlaboratory variability that can impact Magee Equation scores, especially in Ki-67 percentage. In our study, these errors are minimized by reviewing slides by two pathologists. The follow-up time of the study is relatively short. The median follow-up of cases was for 36 months, and seven patients were lost to follow-up. Furthermore, both CTS5 and Magee Equation scores predict the chances of late recurrence after five years, and ER-positive tumors tend to recur late. Hence, a longer follow-up can only validate the predictive potential of these scores. The impact of the Magee Equation score in predicting the response to adjuvant therapy/metastatic potential of the tumor has not been studied extensively in our study. There are certain limitations to the Magee Equation and CTS5 scores in combination to predict recurrence/response. Nevertheless, in an era of rising healthcare spending, alternative strategies should be considered to enhance patient care while reducing healthcare costs.

## Conclusions

Given the rising cost of healthcare, the use of multigene testing should be carefully optimized in breast cancer management. The Magee Equation offers a reliable and economical means of estimating the Oncotype DX recurrence score, supporting treatment planning where genetic testing is not feasible. This study emphasizes a stepwise approach to risk stratification by integrating CTS5 and ME3 scores to better identify patients who may benefit from extended endocrine therapy or chemotherapy. Overall, the Magee Equation 3 serves as a practical, affordable, and effective tool for predicting tumor response and recurrence risk in ER/PR-positive, HER2-negative breast cancers, making it valuable for routine oncologic practice.

## References

[1] O'Sullivan CC, Loprinzi CL, Haddad TC (2018). Updates in the evaluation and management of breast cancer. Mayo Clin Proc.

[2] Sparano JA, Gray RJ, Makower DF (2018). Adjuvant chemotherapy guided by a 21-gene expression assay in breast cancer. N Engl J Med.

[3] Klein ME, Dabbs DJ, Shuai Y, Brufsky AM, Jankowitz R, Puhalla SL, Bhargava R (2013). Prediction of the Oncotype DX recurrence score: use of pathology-generated equations derived by linear regression analysis. Mod Pathol.

[4] Wang C, Chen C, Lin Y (2020). Validation of CTS5 model in large-scale breast cancer population and the impact of menopausal and HER2 status on its prognostic value. Sci Rep.

[5] Tajiri W, Ijichi H, Takizawa K (2021). The clinical usefulness of the CTS5 in the prediction of late distant recurrence in postmenopausal women with estrogen receptor-positive early breast cancer. Breast Cancer.

[6] Lee J, Cha C, Ahn SG (2020). Validation of Clinical Treatment Score post-5 years (CTS5) risk stratification in premenopausal breast cancer patients and Ki-67 labelling index. Sci Rep.

[7] Richman J, Ring A, Dowsett M, Sestak I (2021). Clinical validity of clinical treatment score 5 (CTS5) for estimating risk of late recurrence in unselected, non-trial patients with early oestrogen receptor-positive breast cancer. Breast Cancer Res Treat.

[8] Turner BM, Gimenez-Sanders MA, Soukiazian A (2019). Risk stratification of ER-positive breast cancer patients: a multi-institutional validation and outcome study of the Rochester Modified Magee algorithm (RoMMa) and prediction of an Oncotype DX(®) recurrence score <26. Cancer Med.

[9] Tang G, Cuzick J, Costantino JP (2011). Risk of recurrence and chemotherapy benefit for patients with node-negative, estrogen receptor-positive breast cancer: recurrence score alone and integrated with pathologic and clinical factors. J Clin Oncol.

[10] Turner BM, Skinner KA, Tang P (2015). Use of modified Magee equations and histologic criteria to predict the Oncotype DX recurrence score. Mod Pathol.

[11] Bhargava R, Clark BZ, Dabbs DJ (2019). Breast cancers with Magee equation score of less than 18, or 18-25 and mitosis score of 1, do not require Oncotype DX testing: a value study. Am J Clin Pathol.

[12] Farrugia DJ, Landmann A, Zhu L (2017). Magee Equation 3 predicts pathologic response to neoadjuvant systemic chemotherapy in estrogen receptor positive, HER2 negative/equivocal breast tumors. Mod Pathol.

[13] Saigosoom N, Sa-Nguanraksa D, O-Charoenrat E, Thumrongtaradol T, O-Charoenrat P (2020). The evaluation of Magee Equation 2 in predicting response and outcome in hormone receptor-positive and HER2-negative breast cancer patients receiving neoadjuvant chemotherapy. Cancer Manag Res.

[14] Wang C, Xu Y, Lin Y (2022). Comparison of CTS5 risk model and 21-gene recurrence score assay in large-scale breast cancer population and combination of CTS5 and recurrence score to develop a novel nomogram for prognosis prediction. Breast.

[15] Bhargava R, Clark BZ, Carter GJ, Brufsky AM, Dabbs DJ (2020). The healthcare value of the Magee Decision Algorithm™: use of Magee Equations™ and mitosis score to safely forgo molecular testing in breast cancer. Mod Pathol.

